# 

**Published:** 1881-05

**Authors:** 


ALABAMA DENTAL ASSOCIATION.—The next annual meeting of the Alabama Dental Association will be held in Selma, Ala., on the third Tuesday in (19th day) July, 1881.

The State Board of Dental Examiners will meet at same time and place. Every dentist practicing in the State is expected to be present, as, under the late act of the legislature, everyone practicing dentistry in the State is compelled to have a license from this Board. All dentists are cordlally invited to be present.

T. M. ALLEN, Recording Secretary, Eufaulo, Ala.

We are in hopes that *all* the States will soon enact such laws, and that the profession will see that they are rigidly enforced.—ED.

### HYMENEAL.

"Domestic happiness, thou only bliss
Of Paradise that has survived the fall."

PRICE—ALDEN.—On Wednesday, April 20th, 1881, at St. Andrew's Church, 127th street and 4th avenue, by the Rev. Francis Lobdell, Dr. Sherman B. Price and Miss Jannat Hills Alden, daughter of James M. Alden, both of New York.

---

### NECROLOGICAL.

"Death is the crown of life:
Were death denied, poor man would live in vain."

Richmond, Va., Dr. Charles Macgill, an eminent physician of that

WILL *YOU* PLEASE SEND IN *YOUR* SUBSCRIPTION MONEY *NOW*?

*We take the privilege of deciding what we shall publish, but the author only is responsible for his production.*

We have facilities which enables us to make liberal discounts to our cash subscribers on text and many other books, also Webster's Unabridged Dictionary, the various publications, of Frank Leslie, and Harper, Scribner's Monthly, Lippincott's Magazine, North American Review, Popular Science Monthly, &c., &c., also many of the leading daily and weekly news and pictorial papers.

AN EXTRA

InducemenT

To Subscribers. See the Advertisement of the "Johnson Revolving Book Case in this Journal.

This column will be sold for advertising space six month during the year at *special rates*.

Money is at OUR RISK ONLY when sent by Post Office Money Order, Registered Letter, or Check on Banks in New York City or Baltimore, made payable to Dr. B. M. Wilkerson, 68 N. Charles street, Baltimore, Md.

If you prefer to send check on your private bank, unless located in the cities of Baltimore or New York, please add 25 cents for collection.

